# Expression of Concern for Galdiero et al., “Porins from *Salmonella enterica* Serovar Typhimurium Activate the Transcription Factors Activating Protein 1 and NF-κB through the Raf-1-Mitogen-Activated Protein Kinase Cascade”

**DOI:** 10.1128/iai.00491-24

**Published:** 2024-11-29

**Authors:** American Society for Microbiology

## EXPRESSION OF CONCERN

The American Society for Microbiology (ASM) and *Infection and Immunity* (IAI) are issuing this Expression of Concern regarding the following publication:

Galdiero M, Vitiello M, Sanzari E, D’Isanto M, Tortora A, Longanella A, Galdiero S. 2002. Porins from *Salmonella enterica* serovar Typhimurium activate the transcription factors activating protein 1 and NF-κB through the Raf-1-mitogen-activated protein kinase cascade. Infect Immun 70:558–568. https://doi.org/10.1128/iai.70.2.558-568.2002

ASM and IAI were notified of multiple concerns regarding potential duplication of western blots with this article in IAI by the same corresponding author:

Galdiero S, Capasso D, Vitiello M, D'Isanto M, Pedone C, Galdiero M. 2003. Role of surface-exposed loops of *Haemophilus influenzae* protein P2 in the mitogen-activated protein kinase cascade. Infect Immun 71:2798–2809. https://doi.org/10.1128/iai.71.5.2798-2809.2003

The specific concerns are as follows:

1. In Fig. 2B of the 2002 paper, the SDS-PAGE gels within the “anti-MEK1” and “anti-MEK2” panels labeled “Control” and “S. Ty. LPS (1 ug/ml)” appear identical to SDS-PAGE gels in Fig. 3B of the 2003 paper within the “anti-MEK1” and “anti-MEK2” panels labeled “0’” and “3’” for U937 cells.
2. In Fig. 3 of the 2002 paper, there are several instances of individual bands presented in SDS-PAGE gels that show signs of splicing and flipping along the horizontal axis. These bands also appear identical to some bands presented in Fig. 3 of the 2003 paper.

ASM reviewed the figures and confirmed evidence of apparent duplication in the above-mentioned instances. ASM sent an inquiry to the authors and requested a response from the authors regarding these concerns but due to the amount of time that has passed since publication, the authors could not provide original data for verification and review or provide significant insight into the generation of the figure.

This Expression of Concern is issued to raise awareness of the above-mentioned concerns and will be updated accordingly if new information becomes available. In publishing this Expression of Concern, ASM is following its Publishing Ethics Policies and Procedures and, as a member of the Committee on Publication Ethics (COPE), follows its guidelines in this regard.

